# Comparative study of vestibular projection pathway connectivity in cerebellar injury patients and healthy adults

**DOI:** 10.1186/s12868-022-00702-2

**Published:** 2022-03-22

**Authors:** Byeong Uk Gam, In Hee Cho, Sang Seok Yeo, Jung Won Kwon, Sung Ho Jang, Seunghue Oh

**Affiliations:** 1 Department of Health, Graduate School, Dankook University, 119, Dandae-ro, Dongnam-gu, Cheonan, Chungnam 31116 Republic of Korea; 2 Department of Physical Therapy, College of Health Sciences, Dankook University, 119, Dandae‑ro, Dongnam-gu, Cheonan, Chungnam 31116 Republic of Korea; 3 Department of Physical Medicine and Rehabilitation, College of Medicine, Yeungnam University, 170, Hyeonchung-ro, Nam-gu, Daegu, 42415 Republic of Korea; 4 Department of Physical Therapy, Uiduk University, 261, Donghaedae-ro, Gangdong-myeon, Gyeongju, Gyeongsangbuk-do 38004 Republic of Korea

**Keywords:** Neural connectivity, Cerebellar injury, Vestibular nucleus, Diffusion tensor image

## Abstract

**Objective:**

Cerebellar injury can not only cause gait and postural instability, nystagmus, and vertigo but also affect the vestibular system. However, changes in connectivity regarding the vestibular projection pathway after cerebellar injury have not yet been reported. Therefore, in the current study, we investigated differences in the connectivity of the vestibular projection pathway after cerebellar injury using diffusion tensor imaging (DTI) tractography.

**Methods:**

We recruited four stroke patients with cerebellar injury. Neural connectivity in the vestibular nucleus (VN) of the pons and medulla oblongata in patients with cerebellar injury was measured using DTI. Connectivity was defined as the incidence of connection between the VN on the pons and medulla oblongata and target brain regions such as the cerebellum, thalamus, parieto-insular vestibular cortex (PIVC), and parietal lobe.

**Results:**

At thresholds of 10 and 30, there was lower connectivity in the ipsilateral hemisphere between the VN at the medullar level and thalamus in the patients than in healthy adults. At a threshold of 1 and 10, the patient group showed lower VN connectivity with the PIVC than healthy adults. At a threshold of 1, VN connectivity with the parietal lobe in the contralateral hemisphere was lower in the patients than in healthy adults. Additionally, at a threshold of 30, VN connectivity at the pons level with the cerebellum was lower in healthy adults than in the patients.

**Conclusion:**

Cerebellar injury seems to be associated with decreased vestibular projection pathway connectivity, especially in the ipsilateral thalamus, PIVC, and contralateral parietal lobe.

## Introduction

Balance is a key component that maintains the center of mass within the base of support for ambulation and reduces fall risk [1]. It requires complex integration of the visual, vestibular, and somatosensory systems [2]. In particular, the vestibular system, which is composed of the peripheral vestibular organs in the inner ear, ocular system, and projections of the central nervous system, has relatively low importance for balance in static environments such as horizontal and stable surfaces; however, it is crucial for balance in dynamic environments, where the surface is unstable from tilting and oscillating [3–7].

Vestibular function is controlled by interactions between various brain areas and neuropathways; it affects the balance and vertical position of the head and the body [8]. Studies have reported that vestibular projection pathways were mainly connected with the vestibular nuclei (VN), parieto-insular vestibular cortex (PIVC), cerebellum, and cerebral cortex [9, 10]. The VN, which is located in the pons and medulla oblongata, receives sensory information from eye and head movements as well as body orientation in space to control the movements [11]. The PIVC, which is a core region of vestibular input, contributes to the processing of bodily self-consciousness, estimation of verticality, and integration of visual motion [12]. The cerebellum, which receives vestibular information and projects vestibular information through projection pathways to the VN, contributes to equilibrium [9, 13]. The cerebral cortex contributes to the conscious perception of movement and spatial orientation [11].

Because the vestibular projection pathway is connected to various brain areas, injury to the vestibular system can be accompanied by problems related to balance, spatial orientation, vertigo, and dizziness [14–19]. Moreover, the vestibular projection pathway is connected to the cerebellum [20]. Cerebellar injury can cause not only gait and postural instability, nystagmus, and vertigo but also vestibular symptoms; this is due to the fact that the nodulus of the cerebellum has reciprocal connections with numerous structures in the peripheral and central vestibular networks [13, 14, 21–23]. However, changes in connectivity regarding the vestibular projection pathway after cerebellar injury have not yet been reported.

Recently developed diffusion tensor tractography (DTT), which is derived from diffusion tensor imaging (DTI), has enabled three-dimensional reconstruction and estimation of the microstructural integrity of neural tracts [24–26]. Additionally, DTI enables the projection and reconstruction of functional connectivity and anatomical structures by visualizing water diffusion patterns [25]. Thus, DTI is a useful tool to provide images of the diffusion properties of white matter by quantifying multidirectional connectivity [25]. Studies have reconstructed human neural connectivity in the VN and other brain areas in three dimensions [9, 10, 25]. Therefore, in the current study, we investigated the differences in the connectivity of the vestibular projection pathway after cerebellar injury using DTI tractography.

## Materials and methods

### Subjects

In this study, four stroke patients (three males, one female; mean age 70.75 ± 7.76 years) with cerebellar injury on magnetic resonance imaging (MRI) and 6 control subjects (four males, two females, mean age 30.00 ± 5.66 years) with no history of a neurological or psychiatric disease were recruited for this study at the University Hospital. The inclusion criteria were as follows: (1) first-ever stroke, (2) no traumatic brain injury, and (3) cerebellar injury due to infarction or hemorrhage. All subjects provided informed consent before undergoing DTI and functional evaluations. The study was approved by the Institutional Review Board of Dankook University.

### Diffusion tensor image

DTI data were acquired using a 6-channel head coil on a 1.5 T Philips Gyro scan Intera (Philips, Best, The Netherlands) with single-shot echo-planar imaging. For each of the 32 non-collinear diffusion sensitizing gradients, 67 contiguous slices were collected parallel to the anterior commissure-posterior commissure line. The imaging parameters were as follows: acquisition matrix, 96 × 96; reconstructed matrix, 192 × 192; field of view, 240 × 240 mm^2^; TR, 10,726 ms; TE, 76 ms; parallel imaging reduction factor (SENSE factor) = 2; EPI factor = 49; b = 1000 s/mm^2^; NEX = 1; and a slice thickness of 2.5 mm with no gap (acquired voxel size 1.3 × 1.3 × 2.5 mm^3^) [27, 28].

### Probabilistic fiber tracking

The DWI data were analyzed using the Oxford Center for Functional Magnetic Resonance Imaging of the Brain (FMRIB) Software Library (FSL; www.fmrib.ox.ac.uk/fsl). Affine multi-scale two-dimensional registration was used to correct the head motion effect and image distortion due to eddy currents. Fiber tracking uses a probabilistic image method based on a multifiber model; it was performed in this study by utilizing image routines implemented in FMRIB Diffusion (5000 streamline samples, 0.5 mm step lengths, curvature thresholds = 0.2) [29].

Both contra- and ipsilateral connectivity were defined as the incidence of connection between the VN (on pons and medulla oblongata) and the following target brain regions as well as were determined by whether the results passed through each target brain region: cerebellum, thalamus, PIVC, and parietal lobe. The incidence of connection was counted from the VN (on pons and medullar oblongata) to each brain region. Note that the seed region of interest (ROI) is located at the VN (on pons: Deitets’ and Schwalbe’s nuclei, on medullar oblongata). The fractional anisotropy (FA), mean diffusivity (MD), and tract volume (voxel number) of the projection pathway were also measured.

### Statistical analysis

SPSS software (ver. 20.0; SPSS, Inc., Chicago, IL, USA) was used to analyze the results. The chi-square test was used to determine the significance of differences in the incidences of connectivity in the VN on pons and VN on medullar in patients with cerebellum. The level of statistical significance was accepted for p-values < 0.05.

## Results

A summary of the demographic clinical characteristics of patients with cerebellar syndrome is presented in Table 1. All patients exhibited typical vestibular signs except diplopia: vertigo (n = 4, 100%), ataxia (n = 4, 100%), dysarthria (n = 1, 25%), dysphagia (n = 0, 0%), nystagmus (n = 1, 25%), diplopia (n = 0, 0%), and abnormal facial sensation (n = 1, 25%).

**Table 1:** Demographic of patients with cerebellar injury

No	Sex/age	Vestibular symptoms						
		Vertigo	Ataxia	Dysarthria	Dysphagia	Nystagmus	Diplopia	Abnormal facial sensation
1	M/61	+	+	−	−	−	−	−
2	M/66	+	+	−	−	+	−	+
3	F/75	+	+	−	−	−	−	−
4	M/81	+	+	+	−	−	−	−

+, positive sign; −, negative sign

**Fig. 1:**
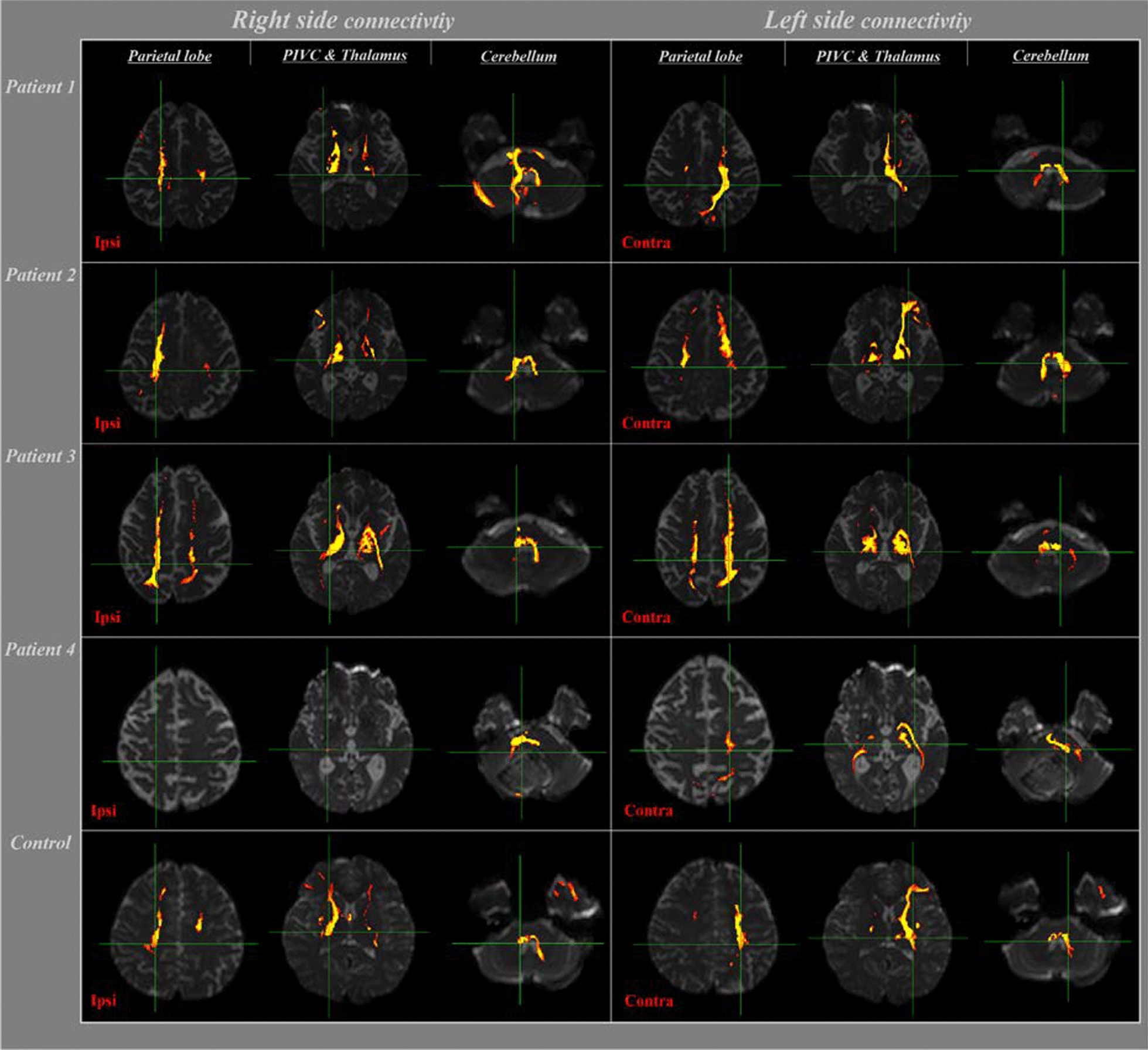
Results of neural connectivity between the VN of pons and vestibular-related areas (parietal lobe, PIVC, thalamus, and cerebellum) in patients with cerebellar injury, at thresholds of 10 streamlines as determined by DTI. The control showed a subject out of six

The reconstruction of VN on pons connectivity is shown in Table 2 and Fig. 1. The ipsilateral connectivity of VN on pons with the target brain regions (cerebellum, thalamus, and parietal lobe) was 100% in healthy adults, regardless of the threshold. In contrast, patients with cerebellar injury showed lower connectivity with the target brain area (cerebellum, thalamus, and parietal lobe). At thresholds of 1, 10, or 30, connectivity with the PIVC steadily decreased in patients (100.0%, 62.5%, and 37.5%, respectively) and in healthy adults (100.0%, 80.0%, and 75.0%, respectively). However, no significant difference was observed between the patients and healthy adults, regardless of the threshold (p > 0.05).

**Table 2:** Comparison of the incidence of connectivity (%) from VN on pons to target brain regions between cerebellar injury patients and healthy adults

Pons level	Threshold (streamline)								
	1			10			30		
Target brain region	Patients	Normal	p	Patients	Normal	p	Patients	Normal	p
Ipsilateral									
Cerebellum	100.0	100.0	1.000	100.0	100.0	1.000	75.0	100.0	0.068
Thalamus	100.0	100.0	1.000	87.5	100.0	0.209	87.5	100.0	0.209
PIVC	100.0	100.0	1.000	62.5	80.0	0.110	37.5	75.0	0.094
Parietal lobe	87.5	100.0	0.209	87.5	100.0	0.209	87.5	100.0	0.209
Contralateral									
Cerebellum	100.0	100.0	1.000	87.5	50.0	0.085	62.5	16.7	0.035*
Thalamus	75.0	100.0	0.068	75.0	58.3	0.444	75.0	33.3	0.068
PIVC	87.5	100.0	0.209	37.5	75.0	0.094	12.5	41.7	0.163
Parietal lobe	87.5	100.0	0.209	75.0	83.3	0.648	62.5	58.3	0.852

PIVC, parieto-insular vestibular cortex

*p < 0.05

At thresholds of 1, 10, or 30, contralateral connectivity of the VN in the pons with the cerebellum steadily decreased in patients with cerebellar injury (100.0%, 87.5%, and 62.5%, respectively) and in healthy adults (100.0%, 50.0%, and 16.7%, respectively). Notably, at a threshold of 30, connectivity with the cerebellum was significantly lower in healthy adults (16.7%) than in patients (62.5%) (p < 0.05). Connectivity with PIVC and parietal lobe also showed decrements with increasing thresholds in patients and healthy adults. It should be noted that at a threshold of 30, connectivity with the parietal lobe was lower in healthy adults (58.3%) than in patients with cerebellar injury (62.5%). However, connectivity at each threshold was not significantly different between the two groups (p > 0.05). Connectivity with the thalamus was 75.0% in patients at thresholds of 1, 10, or 30. In contrast, healthy adults showed lower connectivity with the thalamus at thresholds of 1, 10, or 30 (100%, 58.3%, and 33.3%, respectively).

**Fig. 2:**
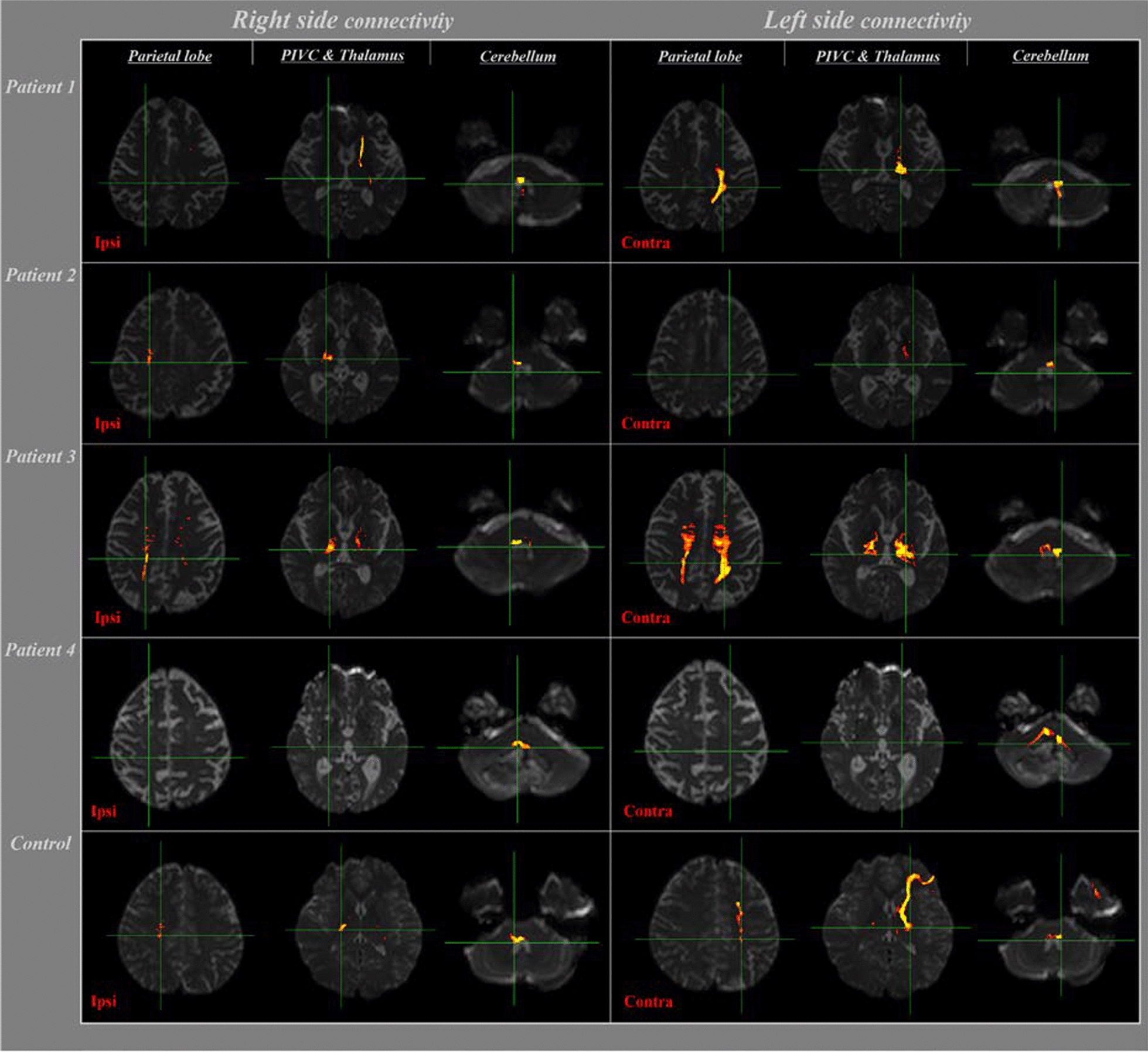
Results of neural connectivity between the VN of medulla oblongata and vestibular-related areas (parietal lobe, PIVC, thalamus, and cerebellum) in patients with cerebellar injury, at thresholds of 10 streamlines as determined by DTI. The control showed a subject out of six

The reconstruction of the VN on the medullary connectivity is shown in Table 3 and Fig. 2. At thresholds of 1, 10, or 30, the ipsilateral connectivity with the cerebellum, PIVC, and parietal lobe steadily decreased in both patients and healthy adults. Notably, at thresholds of 1 and 10, connectivity with the PIVC was significantly lower in patients than in healthy adults (p < 0.05). At thresholds of 1, 10, or 30, connectivity with the thalamus decreased in both patients (75.0%, 50.0%, and 50.0%, respectively) and healthy adults (100.0%, 100.0%, and 91.7%, respectively). At thresholds of 10 and 30, connectivity with the thalamus was significantly lower in patients than in healthy adults (p < 0.05).

**Table 3:** Comparison of the incidence of connectivity (%) from VN on medullar to target brain regions between cerebellar injury patients and healthy adults

Medullar level	Threshold (streamline)								
	1			10			30		
Target brain region	Patients	Normal	p	Patients	Normal	p	Patients	Normal	p
Ipsilateral									
Cerebellum	87.5	100.0	0.209	62.5	50.0	0.582	50.0	25.0	0.251
Thalamus	75.0	100.0	0.068	50.0	100.0	0.006*	50.0	91.7	0.035*
PIVC	50.0	91.7	0.035*	12.5	66.7	0.017*	0.0	25.0	0.125
Parietal lobe	75.0	100.0	0.068	50.0	83.3	0.111	37.5	50.0	0.582
Contralateral									
Cerebellum	62.5	66.7	0.848	37.5	16.7	0.292	0.0	0.0	1.000
Thalamus	62.5	91.7	0.110	50.0	41.7	0.714	12.5	8.3	0.761
PIVC	50.0	83.3	0.111	25.0	8.3	0.306	0.0	0.0	1.000
Parietal lobe	62.5	100.0	0.021*	50.0	33.3	0.456	12.5	8.3	0.761

PIVC, parieto-insular vestibular cortex

*p < 0

At thresholds of 1, 10, or 30, contralateral connectivity of VN on the medulla with all target brain regions (cerebellum, thalamus, PIVC, and parietal lobe) steadily decreased in both patients and healthy adults. Notably, at a threshold of 1, connectivity with the parietal lobe was significantly lower in patients (62.5%) than in healthy adults (100%) (p < 0.05).

## Discussion

In the current study, we investigated the differences in vestibular projection pathway connectivity after cerebellar injury using DTI tractography. We found that at thresholds of 10 and 30, there was lower connectivity in the ipsilateral hemisphere between the VN at the medullar level and thalamus in patients than in healthy adults. At thresholds of 1 and 10, the patient group showed lower VN connectivity with the PIVC compared to healthy adults. At a threshold of 1, VN connectivity with the parietal lobe in the contralateral hemisphere was lower in patients than in healthy adults. Additionally, at a threshold of 30, VN connectivity at the pons level with the cerebellum was lower in healthy adults than in patients. These results suggest that cerebellar injury due to hemorrhage might be associated with alterations in the connectivity of the vestibular projection pathway, especially the thalamus and PIVC in the ipsilateral hemisphere and parietal lobe in the contralateral hemisphere.

Studies have reported that vestibular projection pathways from VN at the level of the pons and medulla are typically connected to the thalamus, PIVC, VN, cerebral cortex, and cerebellum [9, 10, 30]. In 2004, Lee et al. showed that patients with cerebellar infarction presented with isolated vertigo, spontaneous ipsilesional nystagmus, and contralesional axial lateropulsion, without symptoms of cerebellar dysfunction [21]. In 2017, Kim et al. reported that isolated vestibular symptoms were associated with cerebellar injury due to infarctions without other neurologic deficits [13]. Specifically, cerebellar lesions involving the inferior cerebellar peduncle, which include the neural pathway that typically transfers vestibular information to the VN, can lead to isolated vertigo and postural imbalance without other neurological deficits [13, 31]. In 2018, Jang et al. suggested that the VN showed strong connectivity with the cerebellum, thalamus, and vestibular-related brain regions [9]. Our results are consistent with those of the previous studies. The cerebellum receives vestibular inputs and projects through the inferior cerebellar peduncle to the VN [13, 32]. Subsequently, the VN sends vestibular information to the PIVC, which is then processed and integrated with the thalamus [32–35]. When vestibular information is deficient due to cerebellar injury, VN may affect connectivity with the PIVC and thalamus [13, 21]. Hence, cerebellar injury might affect the connectivity of the vestibular projection pathway, especially in the thalamus and PIVC.

In the current study, the VN at the medullar level connectivity with the parietal lobe was lower in patients than in healthy adults, at a threshold of 1 in the contralateral hemisphere. In 1994, Akbarian et al. reported that connectivity was present between the VN and premotor and parietal cortices [36]. Recently, Jang et al. reported the VN connectivity in 37 healthy adults; it has also been reported that the VN showed connectivity with the primary motor cortex (95.9%, 83.8%, and 74.3% at thresholds of 1, 10, and 15, respectively), primary somatosensory cortex (90.5%, 68.9%, and 64.9%), and premotor cortex (87.8%, 52.7%, and 40.5% at thresholds of 1, 10, and 15 respectively) [9]. Our results are consistent with those of previous studies. Thus, cerebellar injury might affect VN connectivity with the parietal lobe.

In the current study, VN connectivity at the pons level with the cerebellum was higher in patients than in healthy adults, at a threshold of 30 in the contralateral hemisphere. Studies have reported that the unaffected hemisphere is associated with neuroplasticity in patients with brain injury [37–39]. In 2010, Kwak et al. demonstrated changes in the corticospinal tract in the unaffected hemisphere in stroke patients using DTI [37]. In 2013, Yeo et al. reported increased fiber volumes of the corticoreticular pathway in the unaffected hemisphere related to the recovery of motor function in stroke patients [38]. In 2016, Jang et al. demonstrated changes in the corticospinal tract in the unaffected hemisphere according to the severity of the corticospinal tract injury in stroke patients [39]. These studies suggest that the change in the neural pathway in the unaffected hemisphere can be regarded as neuroplasticity; therefore, the phenomenon of changes in the unaffected hemisphere can be regarded as a compensation for damage in the affected hemisphere [37–39]. The results of the current study are consistent with those of previous studies. Thus, greater connectivity with the cerebellum in patients than in healthy adults can be regarded as induced neuroplasticity.

The present study has a few limitations. First, it is limited by its small sample size. Second, we only investigated the vestibular projection pathway connectivity in patients with cerebellar injury without clinical evaluation. Third, because DTT cannot discern the direction, the afferent and efferent fibers could not be divided between the VN and target brain regions. Fourth, DTI analysis is operator-dependent; because of fiber complexity and the crossing fiber effect, it may underestimate the fiber tracts. Therefore, to overcome these limitations, in-depth studies as well as studies regarding the clinical application of our results in patients with cerebellar injury are encouraged.

## Conclusion

We investigated the differences in the connectivity of the vestibular projection pathway after cerebellar injury using DTI tractography. We found that cerebellar injury seems to be associated with decreased vestibular projection pathway connectivity, especially in the ipsilateral thalamus, PIVC, and contralateral parietal lobe. Therefore, evaluating the vestibular pathway using DTT in patients with cerebellar injury might be useful for clinical evaluation.

## Data Availability

The datasets generated during and/or analyzed during the current study are not publicly available due to the data containing information regarding individual diseases but are available from the corresponding author on reasonable request.
